# Sparse Sensing and Machine Learning for Rapid Calibration of Defect Detection with rf Atomic Magnetometers

**DOI:** 10.3390/s25226930

**Published:** 2025-11-13

**Authors:** Marek J. Munko, Lucas M. Rushton, Laura M. Ellis, Jake D. Zipfel, Patrick Bevington, Witold Chalupczak, Sergio Lopez Dubon

**Affiliations:** 1 School of Engineering, The University of Edinburgh, Mayfield Road, Edinburgh EH9 3JL, UK; sergio.ldubon@ed.ac.uk; 2 National Physical Laboratory, Hampton Road, Teddington TW11 0LW, UK; lucas.rushton@npl.co.uk (L.M.R.); laura.ellis@npl.co.uk (L.M.E.); jake.zipfel@npl.co.uk (J.D.Z.); witold.chalupczak@npl.co.uk (W.C.)

**Keywords:** non-destructive testing, rf atomic magnetometers, sparse sensing, machine learning

## Abstract

Machine learning algorithms are utilised to improve the speed of both image acquisition and calibration processes needed for defect detection using radio-frequency atomic magnetometers. Sparse sensing is employed, and an average relative error of ≈5% is observed for a reconstructed image based on 1.25% of the sampled pixels when compared to a raster scan over the target object. Additional algorithms demonstrate the viability of image processing to qualify results and adjust experimental parameters required for calibration, leading to an enhancement of image contrast. This presents a first step in developing a tool for fast, arbitrary defect detection.

## 1. Introduction

There is a growing market need for rapid, non-destructive, non-contact methods to detect objects and sub-surface features in asset monitoring and security applications [1]. From the broad portfolio of non-destructive technologies, inductive measurements with radio-frequency (rf) atomic magnetometers present an attractive solution.

**Figure 1:**
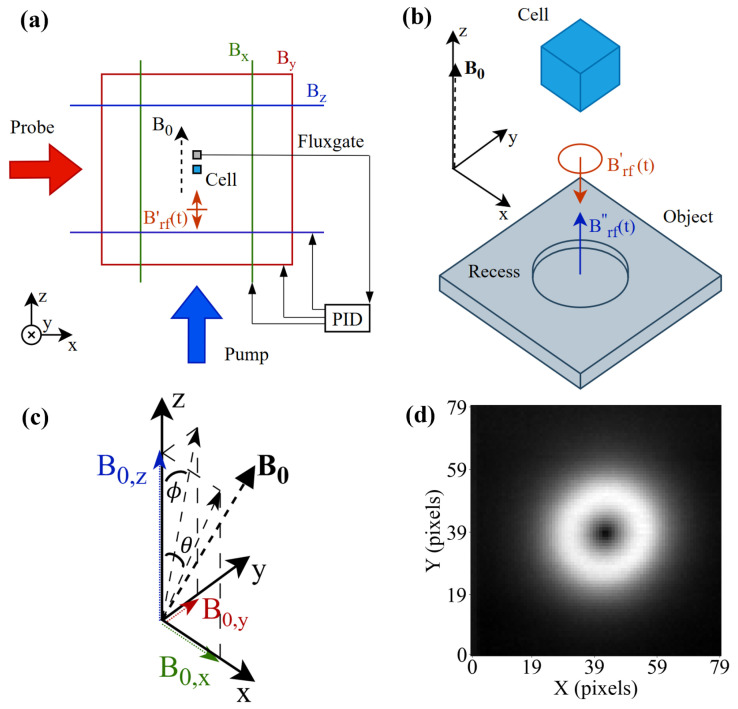
(**a**) Experimental setup of the atomic magnetometer indicating the laser beam direction for the pump and probe beams, as well as the configuration of square Helmholtz coils used in a PID feedback control loop with a fluxgate to define $B_{0}$. (**b**) Inductive measurement setup for a circular recess in a square plate. A primary excitation field $B_{\mathrm{rf}}^{\prime }\left(t\right)$ produces a secondary field $B_{\mathrm{rf}}^{\prime \prime }\left(t\right)$ that is measured by a sensor with a sensitive plane perpendicular to $B_{0}$. If the direction of $B_{0}$ changes, e.g., in (**c**), so does the orientation of the sensing plane. It is assumed that the axis of the $B_{\mathrm{rf}}^{\prime }\left(t\right)$ coil is parallel to $B_{0}$ and centered under the magnetometer. (**c**) Vector diagram showing the angle θ and ϕ that the field $B_{0}$ makes between the *z*-axis and the *x*- and *y*-axes, respectively. (**d**) Signal from a circular recess measured in the self-compensation configuration.

Inductive methods monitor the coupling between an oscillating (primary) magnetic field $B_{\mathrm{rf}}^{\prime }\left(t\right)$ and a target object. The resulting (secondary) magnetic field $B_{\mathrm{rf}}^{\prime \prime }\left(t\right)$ is entirely defined by the object’s electrical conductivity and magnetic permeability [2,3]. Figure 1a shows a generic magnetic induction tomography (MIT) measurement configuration where a sensor measures the total field $B_{\mathrm{rf}}\left(t\right)=B_{\mathrm{rf}}^{\prime }\left(t\right)+B_{\mathrm{rf}}^{\prime \prime }\left(t\right)$. Since magnetic fields do not interact with non-conducting and non-magnetic barriers, inductive methods have the potential to detect concealed objects, such as buried pipes [4] and corrosion under insulation [5,6,7]. Moreover, oscillating magnetic fields have a frequency-dependent propagation depth $\delta \propto f^{-1/2}$ within the target object, enabling detection of sub-surface features, including incomplete weld joints [8] and cavities [9]. To guarantee good penetration of the primary field into the target, this paper considers sensors with operating frequencies < 100 kHz. Frequency-dependent propagation enables one-dimensional information about an object to be obtained at a specific sensor location (referred to as a pixel). To achieve information in three dimensions, additional sensors can be employed at different loci, which is equivalent to the translation of the object detected by a single sensor. The term MIT is used to highlight this ability to describe the entire (3D) extent of an object or defect [2,10].

Traditionally, pick-up coils have been used as the sensing element, as they offer a good sensitivity relative to the simplicity of their system architecture. However, they have some undesirable characteristics, namely, their sensitivity is proportional to the sensor’s operating frequency and volume, and they have a narrow bandwidth (high Q-factor) at a fixed frequency. Additionally, their signal readout comes from sensitive measurements of impedance changes in the coil circuit, which depend on environmental factors, e.g., temperature, demanding frequent sensor calibration [3,11].

### 1.1. Challenge and Motivation

In the following, we identify two critical challenges in performing MIT measurements with rf magnetometers that can be addressed by implementing statistical methods and machine learning (ML) processes.

#### 1.1.1. Bias Field Calibration for Self-Compensation Measurement Geometry

The rf magnetometer is only sensitive to two orthogonal components of the rf field $B_{\mathrm{rf}}\left(t\right)$ perpendicular to the bias field $B_{0}$ [12,13,14]. In the self-compensation configuration, the insensitive axis is aligned with the primary field $B_{\mathrm{rf}}^{\prime }\left(t\right)$ so the sensor only measures the secondary response field $B_{\mathrm{rf}}^{\prime \prime }\left(t\right)$ orthogonal to $B_{0}\;\|\;B_{\mathrm{rf}}^{\prime }\left(t\right)$ [13]. This setup requires precise geometry, where $B_{0}$ is parallel to $B_{\mathrm{rf}}^{\prime }\left(t\right)$ and the object’s surface normal, and the sensor is centered on the $B_{\mathrm{rf}}^{\prime }\left(t\right)$ coil axis. Tilts of $B_{0}$ alter the sensing plane and reduce contrast. For flat, homogeneous objects, no signal appears, but defects or edges break this symmetry, creating measurable components of $B_{\mathrm{rf}}^{\prime \prime }\left(t\right)$ transverse to $B_{0}$. The system is ‘calibrated’ when the angle between $B_{\mathrm{rf}}^{\prime }\left(t\right)$ and $B_{0}$ is zeroed. Imperfections such as surface tilts or misalignment of the $B_{\mathrm{rf}}^{\prime }\left(t\right)$ coil, the object, and the coils that define $B_{0}$ complicate the calibration process.

#### 1.1.2. Measurement Acquisition Time

A critical cost associated with generic non-destructive testing (NDT) is measurement time, e.g., the operator time inspecting difficult-to-reach or remote locations, or process downtime in production settings. Raster scanning with point measurements for full imaging of objects or defects is particularly time-consuming. Sensor arrays can be utilised to perform camera-like measurements, analogous to phased array ultrasonic testing, which has been demonstrated with an atomic sensor for magnetic and microwave field imaging and spatially separated DC magnetic field measurements [15,16,17].

ML can be employed to create an algorithm for rapid calibration by optimising the data acquisition rate and automating the calibration procedure.

#### 1.1.3. Statistical Methods and Machine Learning

Advances in statistical modelling and ML have accelerated the transition towards process automation, leading to a reduction in the time spent to collect and analyse experimental data. The applications of ML can be found in a broad range of non-destructive testing areas. For instance, the applications of ML-based image segmentation have been used to detect and localise defects in pictures of railway track surfaces [18] or step-heating thermography measurements of composite laminates [19]. Notably, ML has been demonstrated in MIT measurements with rf atomic magnetometers for object classification [20]. Image segmentation using manual thresholds, rather than ML, has been demonstrated in clinical MIT measurements, where it was used to separate lesions from the background [21].

Moreover, ML has been successfully employed to reduce total measurement times by enabling reconstruction of a full image from its sparse recordings. Specifically, measurements at optimised sensing locations have been used to reconstruct aircraft wing scans [22], temperature distribution in nuclear plant components [23,24], and full-field dynamic responses of large-scale structures, such as a skyscraper [25].

### 1.2. Aim and Scope

In this work, we aim to investigate the efficacy of an algorithm to optimise measurement locations, thereby reconstructing complete images using a significantly smaller number of measured pixels. We then incorporate ML and image processing to automate the setup calibration process, leading to the creation of a complete process pipeline (Figure 9). Therefore, the purpose of the paper is to discuss the optimisation of applications of an rf magnetometer in a specific non-destructive testing problem, rather than the technical and theoretical details of the sensor operation.

### 1.3. Paper Structure

The paper is divided into the following sections:

**Section 2 —Methods:** outlines the mathematical formulation of the sensing–location–optimisation algorithm and introduces the key concepts of image classification and segmentation implemented in the study.

**Section 3 —Experimental setup:** describes the details of the MIT setup used in the experimental procedure.

**Section 4 —Results:** shows the results obtained in the study, namely the raw full-resolution scans recorded with MIT; the accuracy of reconstructions computed with sparse measurements; the pattern of the optimised measurement locations; the performance of the classification and segmentation algorithms; the state diagram of the automated calibration process; and chosen reconstruction examples.

**Section 5 —Conclusions:** provides the evaluation of the presented method and highlights the key learning outcomes. The section also discusses the suitability of extending the method to related problems and provides direction for future study.

## 2. Methods

First, this section aims to introduce the process used to find the subset of optimised sensing locations and use them to reconstruct a high-resolution system measurement. Subsequently, the necessary image processing techniques are introduced. ML is presented as a means of classifying calibrated samples, and the foundations for creating the direction adjustment vector in the compensation process are discussed.

### 2.1. Optimising Sensing Locations and Full-Resolution Measurement Reconstruction

In this subsection, the key aspects of the sensor optimisation and image reconstruction processes are given. Please refer to the corresponding section in the *Supplementary Material*, where the derivation is described in more detail.

The fundamental idea of compressed sensing is the ability to leverage the sparsity of a real-world signal to reconstruct its full-resolution representation using a limited number of measurements. According to Mahonar et al. [26], any single high-dimensional system measurement, x, can be represented as:

(1) x=ψs,

where ψ is the universal transform basis, such as a matrix of Fourier or wavelet coefficients, and s is a sparse vector. In this way, a sparse signal s can be used to reconstruct high-dimensional information x. However, when historical system measurements are known, the notion of sparsity can be used to optimise individual sensing locations to reconstruct full-resolution signals. The existing system measurements can be used to create a dataset-specific basis by performing singular value decomposition (SVD) [27] on the previously-collected data [22]. Therefore, when *r* sensing locations and a dataset-specific basis $\psi_{r}$ are considered, the following relationship is true [26]:

(2) $$y=C\psi_{r}a,$$

where C is a matrix of sensing locations, y is a vector of sensor measurements at these locations, and a is a vector used for reconstruction. It has been observed that optimal sensing locations can be found when pivoted QR decomposition is performed on $\psi_{r}$. The operation decomposes the input matrix into a product of Q and R, where Q is orthonormal, and R is an upper triangular matrix. When column pivoting is used, i.e., columns are reordered in the computation process to improve QR factorisation, the pivot locations coincide with the optimal sensing locations for the system. Therefore, pivoted QR decomposition can be used to determine the sensing locations C shown in Equation (2). Once C is computed, the measurements of the system, y, can be made at the specific locations. Once y, C, and $\psi_{r}$ are known, a can be determined, as it can be deduced from Equation (2). Finally, for each high-resolution system measurement x, the corresponding reconstruction $x_{\mathrm{rec}}$, can be computed by multiplying $\psi_{r}$ by a.

### 2.2. Image Processing for Self-Compensation Calibration

The calibration method uses two image processing steps to determine: (a) whether the measurement is calibrated (qualification); (b) what fields should be applied to calibrate the measurement (feedback). The former qualifies if the magnetic field $B_{0}$ is aligned with the primary field $B_{\mathrm{rf}}^{\prime }\left(t\right)$ to a satisfactory degree, set by a threshold value defined in Section 4.3.2. For the latter, a direction adjustment vector can be constructed from the measured signal that determines the direction in which $B_{0}$ should be adjusted so that it is aligned with $B_{\mathrm{rf}}^{\prime }\left(t\right)$.

#### 2.2.1. Qualification: Calibrated Image Detection

Determining whether the measurement of a sample is not affected by misalignment of the magnetic field is a classification problem. Although there are many ways to attempt image classification in this case, e.g., by examining the symmetry of the image, recent research trends indicate that ML is a superior classification technique [28,29]. Considering various classification models, such as support vector machines (SVMs), k-nearest neighbour algorithms (kNNs), and artificial neural networks (ANNs), ANNs prove to have the highest accuracy in direct comparisons [30,31]. Among the various ANN architectures, convolutional neural networks (CNNs) are particularly interesting, as they are characterised by the use of convolutional and pooling layers, making them suitable for a range of image processing applications [29,32]. CNNs have become the state-of-the-art in image classification tasks [33].

CNNs typically consist of convolutional layers, which act as trainable filters extracting relevant image features; pooling layers, which reduce the dimensionality of the image; and trainable densely-connected layers, which are commonly used in most ANN types [29,32]. In this work, a combination of these layer types will be used to train a CNN to determine whether the magnetic field alignment has been achieved in a reconstructed sample.

#### 2.2.2. Feedback: Direction Adjustment Vector

In case the model described in Section 2.2.1 classifies the image as misaligned, the direction adjustment vector is created to determine the direction in which the bias magnetic field should be adjusted. The working principle of the method developed in this paper is based on the fact that the image possesses certain features that directly correspond to this direction. One such feature is the location of the low-magnitude pixels, which form a distinctive spot moving about the centre of the scan as the angle of the magnetic field changes. Therefore, the image can be segmented to separate different clusters of low-value pixels, whose centre can be identified through, e.g., centre-of-gravity detection algorithms. This information can be subsequently used to construct the direction adjustment vector.

## 3. Experimental Setup

This paper will only introduce the essential elements of the rf magnetometer as represented in Figure 1a,b and pictured in *Supplementary Material*—a more complete description can be found elsewhere [13,34,35]. The rf magnetometer consists of three main subsystems: the atomic vapour cell, lasers, and detection system.

The atomic vapour cell contains the atomic species of interest, which in this case is caesium. The atoms are polarised into a magnetically sensitive state, and their interaction with $B_{\mathrm{rf}}\left(t\right)$ is monitored optically with probe laser light. The interaction of the pump beam gives the ensemble of atoms a net magnetic moment directed along a tunable static magnetic field $B_{0}$, which defines the rf magnetometer’s operational (Larmor) frequency. A magnetic field rotating in the plane perpendicular to $B_{0}$ at the operational frequency will generate the sensor signal.

Figure 1a shows the set of nested square orthogonal Helmholtz coils that generates $B_{0}$ and is centred around the vapour cell (12 mm cubic glass cell). The coils have side lengths of 1 m, 0.94 m, and 0.86 m, aligned along the *z*, *y*, and *x* axes, respectively. A three-axis fluxgate aligned with the coil axis and located close to the vapour cell monitors the local magnetic field. Each output is used as the error signal for a proportional–integral–derivative (PID) controller that modulates the current driving the corresponding Helmholtz coil in order to stabilise $B_{0}$ to setpoint values $V_{x}$, $V_{y}$, and $V_{z}$ (∝ $B_{0,x}$, $B_{0,y}$, and $B_{0,z}$). The rf field is generated by a 40-turn coil that has dimensions of length 8 mm, outer diameter 4 mm, and inner diameter 2 mm wound on a ferrite core with dimensions of length 10 mm and diameter 2 mm.

For simplicity, the rf magnetometer is stationary and the object is moved beneath the rf coil, which is centred under the sensor. The object’s position is controlled by a Duet 3 Mainboard 6HC that drives two axial pairs of stepper motors coupled to a 3D-printed translation stage. The duet board enables homing and calibrated movement to position utilising G-code commands, and maintains a constant spatial coordinate system. This allows optimised movements between arbitrary pixels, enabling use in the ML processes described.

A calibration process can be employed to find the self-compensation configuration without the presence of the object, such that no signal is generated from a defect within the object or from the object’s edges or surface. However, the introduction of the object leads to signals coming from the object’s edges, tilts in the plate’s surface, or from offsets in the centering of the rf coil and rf magnetometer, all of which can shift the required angle between $B_{0}$ and $B_{\mathrm{rf}}^{\prime }\left(t\right)$ to reach calibration. The two angles θ and ϕ that $B_{0}$ makes with the *z*-axis along the *x*- and *y*-axes, respectively, [shown in Figure 1c], can be used as a coordinate system for calibration. The relevant parameters for this coordinate system and their relationship are described in Table 1. When describing the physical interpretation of the results, the discussion uses intuitive physical units of $B_{0}$ and the angle (e.g., Section 4.1). The setpoint parameter is used in the calibration algorithm and when describing this process (Section 4.2.5 onward). Figure 1d shows an example of the amplitude image recorded for a circular recess after field calibration, and is fully described in Section 4.1.

## 4. Results

This section presents results on three experimental areas. Firstly, the conventional method for calibration is outlined. This is supported by a set of complete scans at various angles between $B_{0}$ and $B_{\mathrm{rf}}^{\prime }\left(t\right)$, which are used to train the ML model used in this study. Next, details for the reconstruction of complete images from reduced numbers of measurement points are presented. Finally, an automation process for calibration is outlined and employed.

### 4.1. Conventional Method for Calibration

In previous studies, a standard procedure to optimise these components is to sweep the angle θ and ϕ, and record an image of an object with a known response. In particular, an engineered defect has been studied to represent a real corroded sample. This takes the form of a circular recess (12 mm diameter) in a square aluminium plate (150 mm × 150 mm × 6 mm) [12,13,35]. Due to the spatial offset between the fluxgate and the rf magnetometer, there is a difference in the required field to null $B_{0,x}$ and $B_{0,y}$ at both sensors. Calibrated settings are observed for $B_{0,x}=0.51\;\mu T$ and $B_{0,y}=0.52\;\mu T$ measured at the fluxgate. These offsets are taken into account when calculating θ and ϕ.

**Figure 2:**
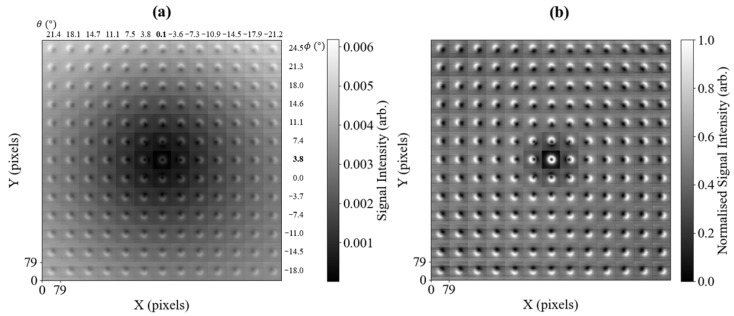
Mosaic of 13×13 amplitude images, each consisting of 80×80 pixels. Images in each column represent a change in the angle θ and each row a change in ϕ, equal to the tilt of $B_{0}$ away from the *z*-axis along the *x*-axis (θ) and *y*-axis (ϕ), respectively. Self-compensation occurs at the central image θ=0.1° and ϕ=3.8°, non-zero due to minor misalignment of the $B_{\mathrm{rf}}^{\prime }\left(t\right)$ coil. Figure (**a**) represents raw data recorded at an operating frequency of 51 kHz ($B_{0}\approx 14.6$ μT), while (**b**) shows the independently-normalised data of each amplitude sub-image in (**a**) (subtracting the smallest value and dividing by the maximum).

Figure 2 shows how the amplitude of the measured signal changes with θ and ϕ. The data in Figure 2a,b are a mosaic of 13×13 sub-images, each with 80×80 pixels, concatenated in a square grid. The concatenated sub-images share the same colour bar in Figure 2a, while the data in each sub-image in Figure 2b were normalised by subtracting the minimum value from each sub-image and dividing by the maximum value. The data in Figure 2a show that the total signal amplitude decreases towards the central sub-image at θ=0.1° and ϕ=3.8°, which is the calibrated self-compensation point. The angles are non-zero to account for minor misalignment of the $B_{\mathrm{rf}}^{\prime }\left(t\right)$ coil. The corresponding phase data for this measurement are shown in the *Supplementary Material* and are not considered further in this calibration analysis.

The benefit of operating at the self-compensation point is the increased measurement contrast, as shown by the normalised sub-images in Figure 2b. Figure 2a shows that at the compensation point, the background signal is minimised. Away from self-compensation, there is a background signal due to the $B_{\mathrm{rf}}^{\prime }\left(t\right)$ component (from the flat surface of the plate and $B_{\mathrm{rf}}^{\prime }\left(t\right)$ along $\hat{z}$) that is perpendicular to $B_{0}$. The recess edge creates a $B_{\mathrm{rf}}^{\prime \prime }\left(t\right)$ component transverse to the surface normal of the plate, directed along the normal of the edge. This component’s direction will change by 360° around the perimeter of the recess. At the self-compensation point, this will show a symmetric, circular ring-like feature. Away from self-compensation, the components of $B_{\mathrm{rf}}^{\prime \prime }\left(t\right)$ from the recess will mix with those from the flat surface of the plate and $B_{\mathrm{rf}}^{\prime }\left(t\right)$. Due to the full rotation of the circular $B_{\mathrm{rf}}^{\prime \prime }\left(t\right)$ edge, there will always be a component of $B_{\mathrm{rf}}^{\prime \prime }\left(t\right)$ that will contribute with the same and opposite sign to $B_{\mathrm{rf}}^{\prime }\left(t\right)$. There are also components that are projected along the insensitive axis. This describes the visible bright and dark lobes, e.g., as seen in the image recorded at θ=0.1° and ϕ=24.5°. As the component of $B_{0}$ projected on the x−y plane rotates by 360° around the self-compensation point, the projection of $B_{\mathrm{rf}}\left(t\right)$ on the sensing plane causes the bright and dark spots in each sub-image to rotate symmetrically around the self-compensation point. Visually, this gives a directional indication of the position of the self-compensation point from each sub-image.

Due to the ≈1 s data acquisition time per pixel [36], the data in Figure 2 took 12.5 days to record, which is unfeasible for normal inspection processes. This dataset can be used as a training dataset for the data reconstruction process, where only a small number of high-priority pixels need to be measured.

### 4.2. Sensing Pixel Location Optimisation

The method presented in Section 2.1 uses training images to optimise the sparse sensing locations (pixels). Subsequently, to increase the credibility of the method’s validation, a new dataset is collected. The images in the validation dataset are sampled at the optimised sensing locations, and reconstructed images are generated. The reconstructed images are compared against the original scans, and the reconstruction accuracy is quantified with chosen error metrics.

#### 4.2.1. Training Dataset

The dataset used for training the algorithm, i.e., finding the optimal sensing locations, consists of all the measurements presented in Figure 2, which were down-sampled from their original resolution of 80×80 pixels to 40×40 pixels by dividing each 80×80 pixel image into 2×2 pixel blocks and finding their average. This way, the data are spatially averaged, matching a 40×40 pixel grid.

The down-sampled dataset, consisting of 169 images, constitutes the training dataset. Considering these images to be arranged in a 13×13 grid (as presented in Figure 2), two of four quadrants are used for the first iteration of the sensing location optimisation. To this end, the two chosen quadrants are rotated and mirrored, so that due to the symmetry of the images, they can imitate the entire 13×13 grid. Using this data as training to optimise the sensing locations, the remaining two quadrants can be used for validation, yielding more credible results than using the same raw data for training and validation.

#### 4.2.2. Validation Dataset

The inspection of the preliminary results showed that the images could be feasibly reconstructed with sparse measurements. However, since all data came from the same scan of the metal plate, it was decided that an independent dataset would be collected. This should verify the resistance of the method to any variations, which naturally occur between different physical measurements, as well as validate the method when data are collected in the resolution of 40×40 pixels right away. Therefore, a dataset of 169 images (following the same 13×13 distribution as presented in Figure 2) has been collected, where each plate scan has the resolution of 40×40 pixels.

#### 4.2.3. Reconstruction Accuracy

For each number of sensing locations considered, *r*, there is an optimal location for each pixel, and together, they form a distinct sensing pattern, affecting the performance of the reconstruction of the full image, $x_{\mathrm{rec}}$. To investigate this, $x_{\mathrm{rec}}$ was calculated for a range of *r* values for each full image, **x**, in the 13×13 image training dataset (Figure 2). The accuracy of each reconstruction is then evaluated using three error metrics, namely mean relative error (MRE), structural similarity index measure (SSIM), and root mean square error (RMSE), to provide a holistic view of the reconstruction quality. The MRE is valuable as it accounts for the reconstruction error across the entire image, relative to the original pixel values. Considering the mean value, rather than single pixels, helps mitigate the impact of individual outliers. The SSIM focuses on the pattern reconstruction, achieving a score of one for identical images. RMSE is considered as it keeps the same units as the original measurements, and unlike the MRE, it emphasises large errors. The implementation details for the error metrics are provided in Appendix A.

**Figure 3:**
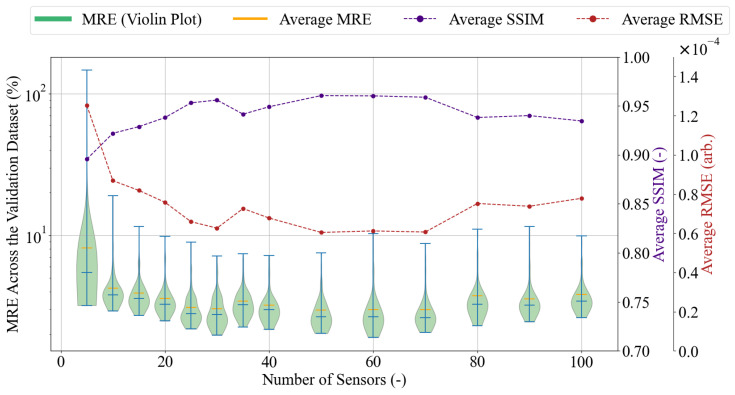
The violin plot representation of the MRE distribution across all 13×13 validation dataset reconstructions, together with the average SSIM and RMSE values for varying numbers of sensing locations *r*. The blue bars in the violin plot mark the distribution’s median and two extrema. Each distribution has a separate vertical axis. The MRE’s vertical axis is on a logarithmic scale to enhance graph readability.

The results presented in Figure 3 show an interesting trend in the MRE variation. The reconstruction error is expected to decrease with an increase in *r*. This is true for a small *r*, namely between 5 and 30 sensing pixels. However, for *r* values between 35 and 100, the averaged MRE ceases to decrease continually and shows minor fluctuations. A certain variation is also apparent in the SSIM and RMSE distributions. Interestingly, all SSIM rises and drops are matched by the opposite RMSE changes, implying that the metrics might be sensitive to similar image features. Although the three metrics aid in the understanding of the reconstruction behaviour, they do not provide an objective criterion for choosing the number of sensors best fitted for the desired application. Therefore, the accuracy of the reconstructions can be further investigated in the context of the presented task through the examination of particular reconstruction cases.

**Figure 4:**
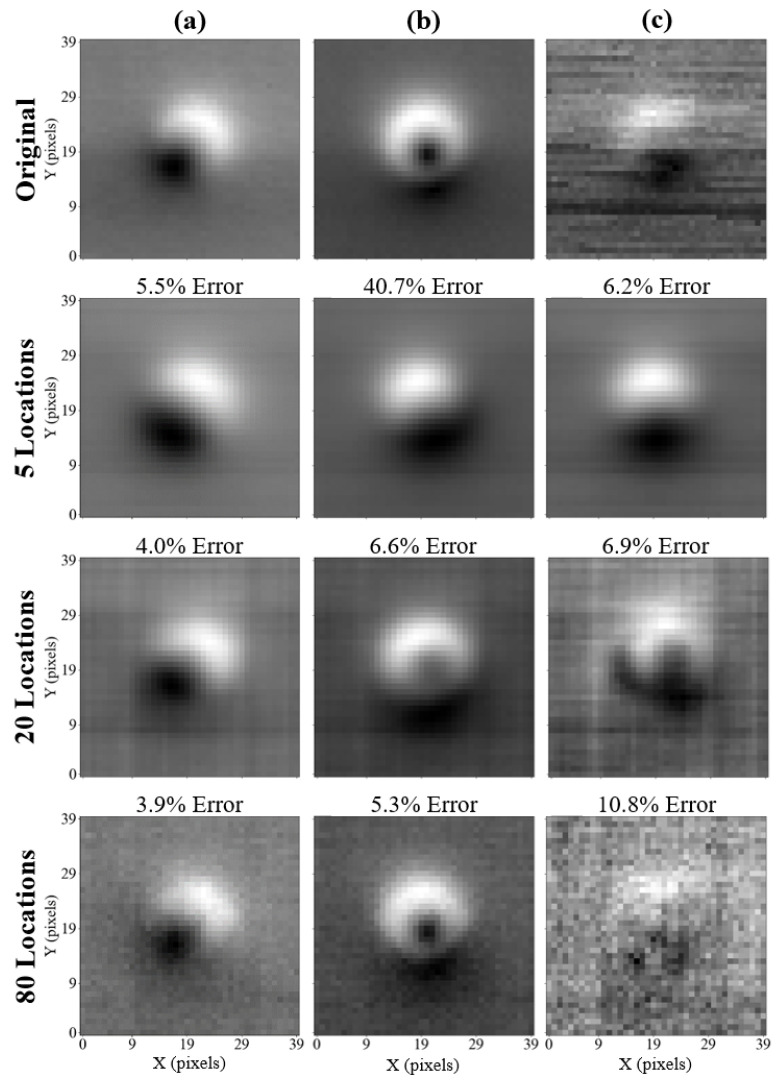
Original (top row) and reconstructed images for chosen samples: (**a**–**c**) measured at angles (θ, ϕ) = (−14.5°, 18°), (0.1°, 7.4°), and (21.4°, 24.5°) respectively. The reconstructions obtained with five, twenty, and eighty data points are presented. Each reconstructed image has the MRE printed above it.

Three cases examined are the following: r={5,20,80}. Respectively, they represent cases in which: the MRE is the highest since the number of sensing locations is the smallest; the MRE decreases with the number of sensing locations; the MRE is relatively high despite a large number of sensing locations. The chosen reconstructions and their MRE values are presented in Figure 4.

The top row in Figure 4 presents the original images in the validation dataset, followed by reconstructions based on the number of sensing locations used in the subsequent rows. The column (a) presents a case in which the expected behaviour is maintained, as the reconstruction error decreases with an increasing number of pixels used for reconstruction. Notably, when fewer pixels are used for reconstruction, the reconstructed image becomes less noisy, e.g., via visual inspection of the images in column (c) in Figure 4.

The images shown in column (b) present a case that disqualifies the use of five sensing locations in a reliable reconstruction method. The feature visible in the original image (the small off-centre black spot) is not reconstructed in the case of five sensing locations. The feature becomes apparent when 20 sensing locations are used. Using 80 sensing locations, a sharp reconstruction of the feature can be achieved, showing the benefit of using many sensing locations in this case.

Considering the images presented in column (c), a considerable amount of noise can be seen in the original sample. In this case, the reconstruction computed with only 5 values shows a clear, denoised image, which can be explained by only the highest-level features being present in the first few singular vectors of the approximated dataset basis used for the sample reconstruction. The reconstruction performed with 20 values shows certain resilience to noise, with the reconstructed feature having the correct location within the image (at the bottom of the main defect). In the case of the reconstruction performed with 80 values, the reconstructed image does not resemble the original one, despite a relatively low error value (10.8%). In this case, a large sensor number leads to erroneous reconstructions by oversampling a noisy measurement, analogous to fitting a high-order polynomial to a noisy dataset.

The number of measured sensing locations is a compromise between measurement time and reconstruction error. As observed, there is no preset criterion based on which the right number of sensing locations could be chosen for the task. Referring again to the accuracy comparison in Figure 3, it can be observed that using 20 sensing locations never results in an MRE greater than 1%. Moreover, the analysis of the selected reconstructions in Figure 4 suggests that the performance of 20 sensors is acceptable, as the characteristic features are preserved. Therefore, it has been decided that 20 sensing locations will be used in the further study and the development of the automated calibration method. Making this choice at the current stage of the experimental procedure is subject to certain uncertainty, and the number of sensors can be increased or decreased depending on the success of the final calibration performance.

#### 4.2.4. Optimal Sensor Distribution and Its Meaning

The 20 optimised sensing locations represent 1.25% of all measured values in a 40×40 pixel scan and therefore the measurement time. The measurement time for a reconstructed image, including sample movement time for the 20 optimised sensor locations, is 24 s, which is defined by the 1 s rf resonance scan time. It should be noted that tailored sensing is influenced by random noise, e.g., highlighted by the additional dark region to the left of the central feature in column (c) for 20 locations in Figure 4.

**Figure 5:**
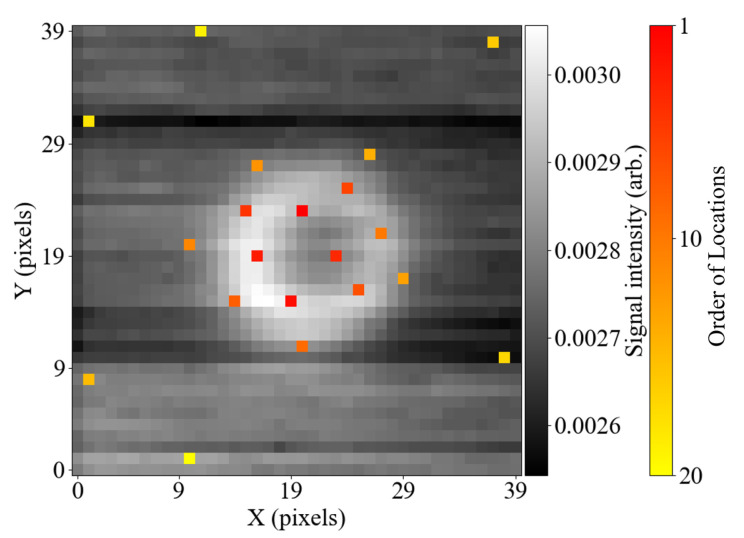
Twenty optimised sensing locations displayed over the averaged map from the collected dataset. The colour scheme of the sensing locations highlights the order in which the locations have been computed.

Figure 5 shows the optimised twenty sensing locations, displayed on top of the average of all images within the training dataset. Moreover, the order in which the locations have been computed in the pivoted QR factorisation process is highlighted using the colourbar shown. A certain physical meaning can be associated with this order when the computation process is considered in detail. The QR factorisation algorithm used in this work is based on the LAPACK library [37], where the first pivot location is chosen to be the pixel that has the largest $L^{2}$ (Euclidean) norm across all collected samples. Subsequently, an orthogonalisation technique, such as the Gram–Schmidt projection [38], can be used to orthogonalise the remaining entries with respect to the chosen vector. The following pivot locations are then selected in an iterative process, where the pixel with the largest $L^{2}$ norm is chosen, and the remaining entries are orthogonalised with respect to it.

Orthogonalising vectors ensures that the redundancy of information is minimised, while selecting an entry with the largest norm helps identify the most important image features. It can be observed in Figure 5 that the locations of the pixels in the image are likely not random, but rather follow an intuitive pattern. The sensing locations that were computed earlier mark the most critical parts of the scanned feature and are located around the defect’s edge circumference. The locations computed later scan the amplitude of the background signal and are evenly spaced around the sample.

#### 4.2.5. Image Reconstruction

The success of reconstructing images by sub-sampling the training data has been shown. In this subsection, we instead perform the reconstruction by only measuring at the optimised sensing locations. In this way, there is no complete image to compare the reconstructions against; however, we can use Figure 2 to assess whether the changes in the applied transverse magnetic fields are accurately reflected in the reconstructions.

**Figure 6:**
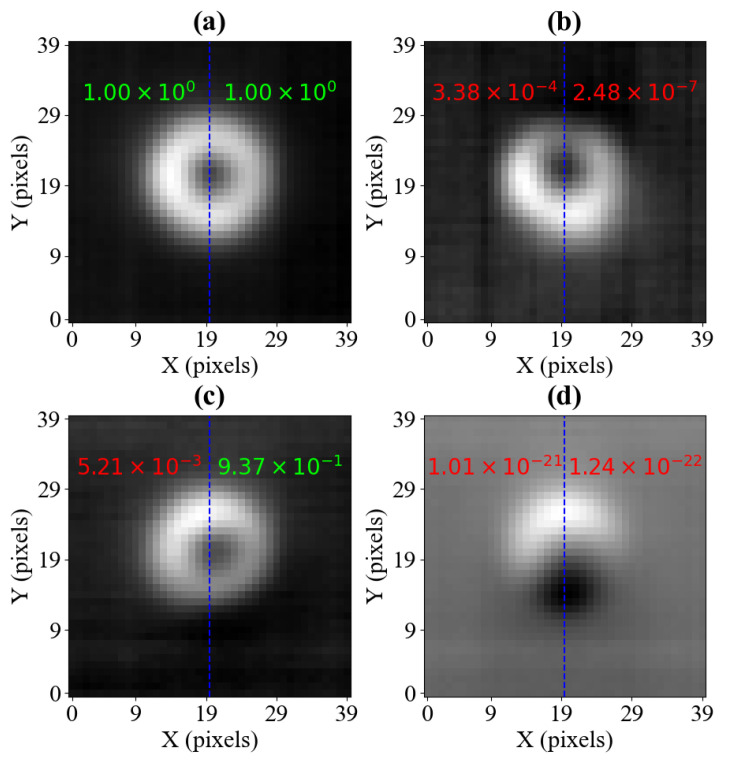
Four images reconstructed from the pixel values collected at twenty pre-set sensing locations, as shown in Figure 5. The $V_{x}$ setting is 0.053 V for all measurements, and $V_{y}$ is set to 0.151 V for (**a**), 0.101 V for (**b**), 0.201 V for (**c**), and 0.401 V for (**d**). The neural network classification results for the sampled images are also included, with the inference score printed on each half-image input to the model. The half images are separated by a dashed blue line. Red scores show off-centred samples, and green scores show samples classified as centred, assuming a classification threshold value of 0.9.

Figure 6 shows the reconstructed images for a fixed $B_{0,x}$ and a series of $B_{0,y}$ strengths, parameterised by the setpoint voltage values $V_{x}$ and $V_{y}$, respectively. The sensing locations superimposed on the reconstructed images are shown in Appendix B. Figure 6a represents the pixel set recorded for $V_{y}=0.151$ V, which reflects calibrated settings, equivalent to the centre of Figure 2. The set illustrated in Figure 6b represents a −0.05 V change in the value of $V_{y}$, in agreement with expectation, and shows a shift of the characteristic black spot upwards. The set in Figure 6c was recorded for a $V_{y}$ value shifted by +0.05 V in the opposite direction from calibrated settings, resulting in the small shift of the characteristic black spot downwards. The set illustrated in Figure 6d was recorded for a much larger $V_{y}$ value, 0.401 V, which results in a very noticeable shift of the black dot downwards. It is worth stressing that the tendencies visible in the reconstructed images agree with those present in Figure 2, supporting the discussed method. Additionally, the cases represented in Figure 6b–d demonstrate that the model is able to interpolate between images, distinguishing features in images with a smaller voltage step than is used in the training dataset, which has voltage steps ≈ 0.1 V between measurements.

### 4.3. Implementation of the New Calibration Method

Following the introduction of the concept of the model in Section 2.2.1, a CNN is trained to determine whether a reconstructed sample represents the calibrated settings. This qualification process is outlined in Section 4.3.1 and Section 4.3.2. Subsequently, the method for the direction adjustment vector is presented in Section 4.3.3, which is termed feedback. Finally, this feedback cycle is outlined in a calibration process flowchart.

#### 4.3.1. Qualification: Training Dataset

In this subsection, we describe the dataset used to train the CNN capable of classifying the reconstructed image as either ’centred’ or ’misaligned’. The symmetry of a reconstructed image is its key feature and indicates the quality of alignment of the bias magnetic field. In the results presented here, this results from the radial symmetry of the defect and its image recorded in calibrated settings [35].

The classification model is intended to work with images reconstructed directly from 20 system measurements. However, collecting a new grid of images (akin to the one presented in Figure 2) reconstructed from sparse system measurements would require additional resources (available measurement time), as image reconstructions would need to be computed for different $V_{x}$ and $V_{y}$ settings. Therefore, the model is to be trained on the same dataset as the sensing location optimisation algorithm, aided with suitable image segmentation. The only addition to the dataset is a set of measurements collected for the calibrated setting (in this case, 203), which creates a substantial dataset of centred images, fighting the imbalance between the ‘centred’ and ‘misaligned’ classes, which is crucial for training a classifier.

Artificial noise is applied to the collected dataset so that the images better resemble the actual reconstructions, characterised by blurred features. To this end, Gaussian blurring is applied to change the pixels’ intensity, and the heuristic approach is taken to determine the necessary noise levels. Through visual inspection, the noise level with a standard deviation of 1.5 is considered to make the images in the training dataset resemble the actual measurement reconstructions. However, given the failure of the CNN’s first training iteration to classify reconstructed samples correctly, the Gaussian standard deviation is increased to 2.5 to make the training more robust. Therefore, alongside the original images, the blurred samples with the standard deviation values of 1.5 and 2.5 are used. Each image is normalised, so that its pixel values vary from 0 to 1, which is a common practice in ML. The classification model should only be sensitive to the recorded patterns (the relative values in the image), and not the absolute value of the measured response.

Initial training attempts have shown poor results on samples that were recorded for a nearly-aligned magnetic field, as such samples were classified as aligned, e.g., like those presented in samples (b) and (c) in Figure 6. Therefore, instead of using a 40×40 pixel input, a 40×20 image is used, taking advantage of the presence of a vertical symmetry axis for a calibrated sample. Half-images have been used to train the CNN (the right-hand side halves are mirrored horizontally to have the same orientation as the left-hand side), resulting in significantly improved classification performance.

#### 4.3.2. Qualification: Image Classifications

The details of the CNN’s training procedure and the final architecture are presented in Appendix C. The performance of the CNN is showcased in Figure 6, where four different reconstructed samples are displayed along with their respective classification results.

Among the four images, only the image shown in Figure 6a was recorded with the calibrated settings. The output of the CNN on which the classification is based is a single continuous value between 0 and 1. If the output value is 1, the model has absolute confidence that a 40×20 pixel input (representing half of the reconstructed image) comes from an image recorded with calibrated settings. When the output is 0, the model has certainty that the setup is not properly calibrated. The values in between represent cases when the model cannot classify the sample with total confidence, and in practice, a manual threshold needs to be chosen to distinguish between ’centred’ and ’misaligned’ classifications.

The classifications in Figure 6 show a spectrum of possible outcomes. The model’s output for sample (a) is equal to 1.00 when rounded to three significant figures, meaning that the sample is clearly classified as calibrated. Considering the image in Figure 6b, whose misalignment is relatively minor, the model correctly classified both halves as incorrectly calibrated. The image in Figure 6c presents evidence that the separate classification of the two image halves brings considerable benefits. Namely, the right-hand side of the image exceeds the threshold of 90% set in this example, suggesting that the sample is centred. However, the left-hand side reaches a significantly lower score, which indicates that the sample is misaligned. Therefore, by setting the condition that both scores need to exceed the classification threshold to assume that the setup has been correctly calibrated, the image in Figure 6c is classified as misaligned, which agrees with our background knowledge about the measurement. The image in Figure 6d proves that the model also works properly when the halves are visibly off-centre, with the output scores being equal to zero to three significant figures. An issue with this method is that it requires the circular image features to be centred.

#### 4.3.3. Feedback: Constructing the Direction Adjustment Vector

As mentioned in Section 2.2.2, the construction of the direction adjustment vector can take advantage of some of the characteristic features of the recorded images. One such feature is the cluster of low-value pixels, whose location is dependent on the angle between the bias and primary magnetic fields, θ and ϕ.

The direction adjustment vector is constructed between two points, one of which is the centre of the image, and the other depends on the location of the characteristic feature. Therefore, the first step in finding the direction adjustment vector is to identify the location of low-value pixels (which, for the colour map used in this work, are represented by darker shades). The pixels are considered ’low-value’ based on a manually-chosen threshold. In this work, a threshold of 3% is used. The identified pixels are then grouped into separate clusters, where every pixel shares at least one edge with any other pixel in the same cluster. Subsequently, the largest cluster is identified, as it is most likely to be the characteristic feature of interest. Since the shape of the cluster can be irregular, the most robust way of determining its centre is by finding its centroid.

**Figure 7:**
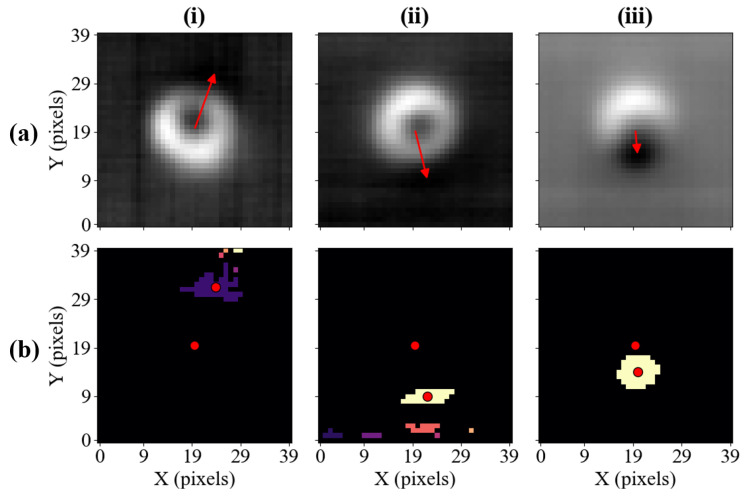
(**a**) The images reconstructed using sparse measurements with the overlying direction adjustment vector for images. (**b**) The representation of segmented images, where all points whose magnitudes are below a certain threshold are coloured. Points belonging to the same cluster have the same shade. One of the red dots shown marks the centre of the image, while the other one marks the centroid of the largest cluster of points, constituting the location of the vector’s head.

Row (a) in Figure 7 shows the direction adjustment vector constructed for three images. In row (b), pixels with values smaller than 3% of the maximum amplitude are highlighted, and each cluster is marked with a different colour. Moreover, the coordinates of the vector’s tail and head are marked, providing context for how the corresponding direction adjustment vector has been constructed. Samples (i) and (ii) have been reconstructed for a $V_{y}$ setting of 0.101 V and 0.201 V, respectively, representing the offset of 0.05 V relative to the calibrated position in both cases. The low-value pixels in both cases form multiple clusters and lie outside of the circumference of the main defect. In contrast, sample (iii) has been recorded for a significantly larger $V_{y}$ setting ($V_{y}$ = 0.401 V), and the low-value pixels form one, nearly circular cluster of points.

**Figure 8:**
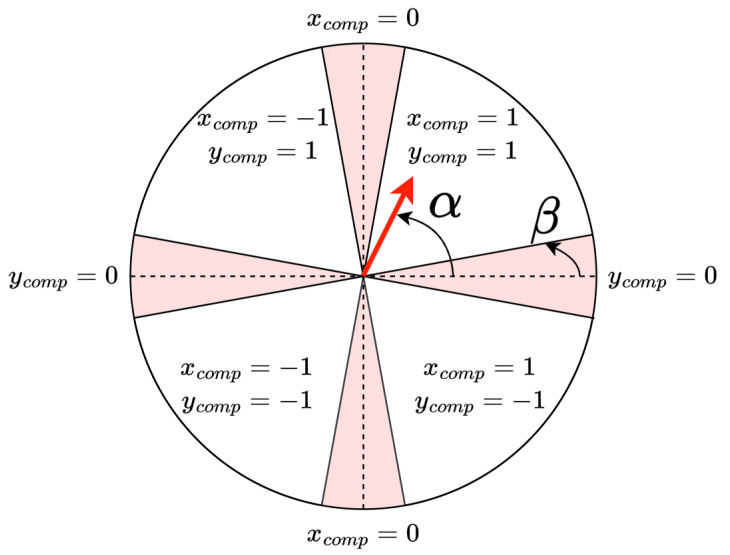
The graphical representation of how $x_{\mathrm{comp}}$ and $y_{\mathrm{comp}}$ values are determined based on the α angle, shown in the figure for a sample direction adjustment vector (in red). The β angle is used to determine the areas where the vector is considered to have solely one direction component.

The property of interest for the calibration process is the angle of the vector, α, which can be used to determine the change in the bias magnetic field. The α angle is measured relative to the horizontal axis, as presented in Figure 8, and it can take values between 0° and 360°. Depending on the value of α, it can be determined whether the compensation should occur in the north or south direction along the vertical axis, and in the east or west direction along the horizontal axis. This way, the corresponding *x*-component ($x_{\mathrm{comp}}$) and *y*-component ($y_{\mathrm{comp}}$) are established, where $x_{\mathrm{comp}}$, $y_{\mathrm{comp}}\in \left\{-1,0,1\right\}$.

Moreover, given the level of noise present in the process, we define a second parameter β, which sets a range interpreted as each of the cardinal directions. Specifically, if the direction adjustment vector points along a cardinal direction within the angular error set by β, the perpendicular directions will not be updated for that loop. This ensures the calibration converges quickly. The necessity for the β angle to ensure the calibration converges quickly is evident considering the samples presented in Figure 7. The bias field of each sample has been offset solely along the vertical axis; therefore, it is also desired that the compensation takes place only along this axis, despite the horizontal component being present in each direction adjustment vector.

#### 4.3.4. Calibration Process Flowchart

Having demonstrated the ability to reconstruct images from twenty pixel values recorded at pre-set locations with satisfactory accuracy, as well as having developed a methodology to reliably assess the calibration of the system and determine the adjustment direction, it is possible to combine these steps into an automated process. It is assumed in the process that the sample of interest has been scanned for a number of bias field settings, and a representative dataset has been collected and used to optimise the sensing locations. Factors such as the physical alignment of the sample and the parameters of the primary field can be altered, as they will be compensated for in the calibration process. Possible alterations and extensions to this method are described in Section 5.

**Figure 9:**
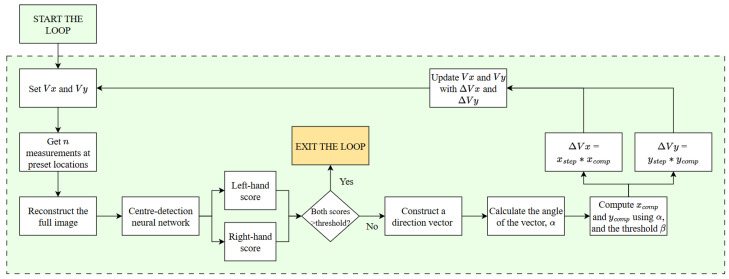
The flowchart for the magnetic field centring process over a fixed sample. The diagram presents the implementation-ready embodiment of the method developed in this work.

The calibration process should begin with a choice of initial $V_{x}$ and $V_{y}$ values, which define the direction of the bias field. The values can be derived from a historical test or come from a different educated estimate. Subsequently, the first measurement can begin by scanning the specimen at pre-set locations, determined in the optimisation process. Once the reconstruction is computed from the recorded values, the image is split into two halves, as outlined in Section 4.3 and passed through the pre-trained neural network. If both inference scores exceed the set threshold, the calibration process can be considered successful, and the loop can be exited. Otherwise, the calibration is attempted, in which case, a direction alignment vector is constructed for the image. Based on its angle, α, and the β setting, $x_{\mathrm{comp}}$ and $y_{\mathrm{comp}}$ values are chosen. They are then used to compute the change in the value of $V_{x}$ and $V_{y}$, denoted as $\Delta V_{x}$ and $\Delta V_{y}$, respectively. The magnitude of the step can be adjusted through the parameters $x_{\mathrm{step}}$ and $y_{\mathrm{step}}$ in the *x*- and *y*-directions, respectively. Finally, $\Delta V_{x}$ and $\Delta V_{y}$ (which can be positive or negative) are added to $V_{x}$ and $V_{y}$, and another measurement can begin with the updated parameters. The loop is set to continue from this point until the neural network classifies both halves of the reconstruction as ’centred’. The calibration process is presented as a flowchart in Figure 9.

The process presented in Figure 9 follows closed-loop control, which ensures that, through iteratively adjusting the chosen $V_{x}$ and $V_{y}$ values, the final voltage settings result in a centred image that is radially symmetric. The speed of convergence depends on the $x_{\mathrm{step}}$ and $y_{\mathrm{step}}$ values. However, the step size must not be too large, as this can lead to instability near convergence, where repeated overshooting exceeds the confidence region, unless additional logic for dynamic step adjustment is introduced. The process outlined here has been successfully employed on our experimental set-up, with the paths taken to calibrate the system shown in Appendix D.

#### 4.3.5. Discussion

There are several limitations to the methods presented here, some of which can be mitigated with further work or when optimised for specific needs. Firstly, the method presented requires a training dataset with pixels corresponding to spatial coordinates on a sample. If the sample were to change relative to the training data, e.g., it would have a different shape or size of recess, then the reconstruction would not represent the physical object. However, more samples could be included in the training dataset, and this would only have to be completed once. Features of the same shape but different sizes should be accurately reconstructed by equivalently scaling the spatial step between pixels. Additionally, spatial changes in object position relative to pixel location will impact the reconstruction; however, simple, low-resolution image centring could be carried out regularly. Secondly, in this proof-of-principle demonstration, several parameters are manually set, i.e., ’low-value’ pixel threshold for constructing the direction adjustment vector (Section 4.3.3); CNN output classification threshold to determine if a half-image comes from a calibrated measurement; the angle β to define when only one component, $V_{x}$ or $V_{y}$, is changed; and a fixed size of the step change in $V_{x}$ and $V_{y}$. Many of these threshold values could be dynamically changed based on initial condition measurements, e.g., step size $V_{x}$ and $V_{y}$ based on the rate of change between relative images.

In essence, the method discussed in this paper significantly speeds up measurement time but is a lossy process, which could potentially miss or hide features that are present, or distort the image and provide erroneous features. To mitigate this, an additional sampling process could be added that performs higher-resolution spatial scans or additional reconstruction on areas identified as suspect. This could include measurements that have correctly identified an unexpected feature in quality control-type measurements, highlighting the need for further testing. Finally, the quality of the reconstructions could be improved through interpolation or ML-based techniques.

## 5. Conclusions

Raster scans, such as those required to record images in Figure 2, define the time duration of MIT measurements involving rf atomic magnetometers. In this paper, a method of sparse sensing was presented, where only a small subset of optimised sensing locations is required to reconstruct the full image. Specifically, it was demonstrated that the recording of 20 pixels, amounting to 1.25% of the complete measurement, can facilitate a reconstruction of the full image with around 5% relative error. Raster scanning was used to generate data for optimising the sensing locations. However, once this was completed, subsequent measurements based on reconstruction were performed in a fraction of the required time.

The 13×13 mosaic of images in Figure 2 is a useful calibration procedure when performing inductive measurements with an rf atomic magnetometer. It identifies the self-compensation geometry when the insensitive axis of the magnetometer (directed along $B_{0}$) is steered parallel with the excitation field $B_{\mathrm{rf}}^{\prime }$, which enables high-contrast detection of defects and objects. However, since there is directional information contained within a single high-resolution sample scan, it is not necessary to record the full 13×13 image grid. We presented a robust CNN model to determine whether the image recorded is in the self-compensation configuration, and an image-segmentation-based method to obtain the sign of change to be applied to an uncalibrated sample. These steps can be integrated into a closed-loop control system, allowing for automated self-calibration from an arbitrary starting point.

This work concentrated on ML-aided detection of a feature with a well-defined shape. The method benefited from the symmetry of the analysed image, reflecting the symmetry of the studied feature. It can be envisaged that the approach presented can be readily extended to scenarios where observed defects could be categorised as realisations of pre-defined classes of defects with different types of symmetries. In a general sense, this is the first step towards ML-enhanced defect detection with an rf atomic magnetometer based on MIT.

## Figures and Tables

**Table 1:** Relation among PID setpoint, field, and compensation angle.

PID Setpoint	Bias Magnetic Field Component	Angle with z-Axis
$V_{x}$	$B_{0,x}$	$\theta =\tan^{-1}\left(B_{0,x}/B_{0}\right)$
$V_{y}$	$B_{0,y}$	$\phi =\tan^{-1}\left(B_{0,x}/B_{0}\right)$
$V_{x}$	$B_{0,z}$	-

**Figure A1:**
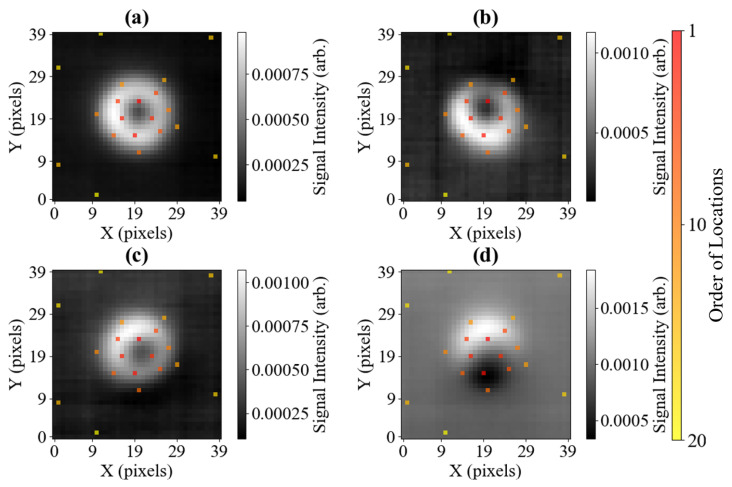
Four images reconstructed from the pixel values collected at twenty pre-set sensing locations. The sensing locations are marked with a colour scheme, reflecting the order in which they have been computed. The $V_{x}$ setting is 0.053 V for all measurements, and $V_{y}$ is set to 0.151 V for (**a**), 0.101 V for (**b**), 0.201 V for (**c**), and 0.401 V for (**d**).

**Figure A2:**
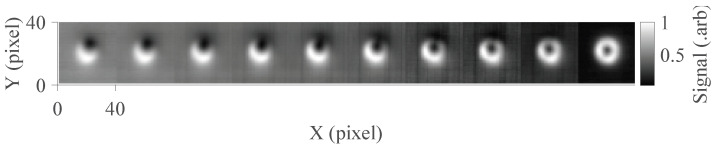
Mosaic of 1×10 normalised amplitude images for a full calibration process, starting form an angle of (θ,ϕ)=(2.2,−3.0)°) to compensation at (−0.7,−3.7)° in 10 steps. The step between each measurement is set to $V_{x}=V_{y}=\pm 0.02$ V, using a model that is trained on measurements with steps of 0.1 V. The images from left to right are the steps described in Table A2.

## Data Availability

The data that support the findings of this study are available from the corresponding author upon reasonable request.

## Supplementary Materials

The following supporting information can be downloaded at https://www.mdpi.com/article/10.3390/s25226930/s1, Figure S1: (a) Image of the phase response for the calibrated measurement and for (b) the full calibration training data set, shown in Figure 2b in the main text.; Figure S2: Images of the experimental setup with scanning rig showing; Figure S3: (a) A chosen 80 × 80 pixel sample from the original dataset. (b) The 40 × 40 representation of the sample shown in (a) following a downsampling process.

## Appendix A. Error Metrics

In this section of the Appendix, the expressions for the three error metrics used in this work are given.

### Appendix A.1. Mean Relative Error

The computation of the MRE between a sample x and its reconstruction $x_{\mathrm{rec}}$ is implemented using the following expression:

(A1) $$\mathrm{MRE}=\frac{\mathrm{mean}(\left|x_{\mathrm{rec}}-x\right|)}{\mathrm{mean}(\left|x\right|)}.$$

### Appendix A.2. Structural Similarity Index Measure

The expression for SSIM is given by [39]:

(A2) $$\mathrm{SSIM}=\frac{\left(2\mu_{x}\mu_{x_{\mathrm{rec}}}+C_{1}\right)\left(2\sigma_{xx_{\mathrm{rec}}}+C_{2}\right)}{\left(\mu_{x}^{2}+\mu_{x_{\mathrm{rec}}}^{2}+C_{1}\right)\left(\sigma_{x}^{2}+\sigma_{x_{\mathrm{rec}}}^{2}+C_{2}\right)}$$

where $\mu_{x}$ and $\mu_{x_{\mathrm{rec}}}$ are the mean of x and $x_{\mathrm{rec}}$, respectively; $\sigma_{x}$ and $\sigma_{x_{\mathrm{rec}}}$ are their standard deviations; $\sigma_{xx_{\mathrm{rec}}}$ is their covariance; and $C_{1}$ and $C_{2}$ are constants. According to [39], the values of constants are calculated as:

(A3) $$C_{1}={\left(K_{1}L\right)}^{2};C_{2}={\left(K_{2}L\right)}^{2}$$

where *L* is the dynamic range of values, and $K_{1}$ and $K_{2}$ can be set to 0.01 and 0.03, respectively [39]. The dynamic range for the problem considered is taken to be the difference between the maximum and minimum values in the training dataset, evaluated to be 0.006083.

### Appendix A.3. Root Mean Square Error

The RMSE is computed using

(A4) $$\mathrm{RMSE}=\sqrt{\mathrm{mean}({\left(x-x_{\mathrm{rec}}\right)}^{2})}.$$

## Appendix B. Reconstructed Images Superimposed with the Sensing Locations

In Figure A1, the sensing locations are superimposed on the reconstructed data from Figure 6.

## Appendix C. Convolutional Neural Network

The structure of the model, including the type and size of layers, as well as the associated activation functions, is presented in Table A1. The training is performed using a learning rate of 0.001 and an 80:20 split between the training and test data, respectively. The model is trained for fifteen epochs and reaches 100% accuracy on the test dataset. The size of the file required to run its inference, containing the structure and weights of the model, is 2.37 MB, and as shown in the following sections, the model also performs satisfactorily in real-life conditions.

The model’s structure has been developed using a set of layers most commonly found in CNNs. Considering its high accuracy and its size, which poses no challenge to its storage or deployment, given the computational resources available, the model is incorporated into the processing pipeline. However, it can also be noted that although the trained CNN provides a satisfactory solution, many other models, characterised by different parameters and architecture types, can be developed to tackle the same classification problem.

**Table A1:** Neural network structure.

Layer (Type)	Number	Kernel Size	Activation
Conv2D	32	(3, 3)	ReLu
MaxPooling2D	-	(2, 2)	-
Conv2D	64	(3, 3)	ReLu
MaxPooling2D	-	(2, 2)	-
Conv2D	64	(3, 3)	ReLu
Flatten	-	-	-
Dense (fully-connected)	64	-	ReLu
Dense (fully-connected)	1	-	Sigmoid

## Appendix D. Full Calibration

Figure A2 shows a demonstration of the automated calibration process detailed in Section 4.3.4. The measurement starts at a point several steps away from the calibration point, with all $V_{x}$ and $V_{y}$ (θ and ϕ) values listed in Table A2. This procedure demonstrates two things. Firstly, the original training dataset has a step of $V_{x}=V_{y}=\pm 0.1$ between each measurement, while in this demonstration, it is limited to $V_{x}=V_{y}=\pm 0.02$. Secondly, only a limited number of steps were shown in this example for ease of display; however, the process is successful when started outside the training range. Together, these demonstrate the flexibility of this approach, which can handle measurements not included in the original training and make successful predictions for calibration. The full calibration sequence took 240 s.

**Table A2:** Field angles for full calibration sequence.

Step	Applied Field $\left(V_{x},V_{y}\right)$ (V)	Angle (θ,ϕ) (°)
0	(0.11, −0.03)	(2.2, −3.2)
1	(0.09, −0.01)	(1.5, −2.2)
2	(0.09, 0.01)	(1.5, −1.5)
3	(0.09, 0.03)	(1.5, −0.7)
4	(0.09, 0.05)	(1.5, 0.0)
5	(0.07, 0.07)	(0.7, 0.7)
6	(0.05, 0.09)	(0.0, 1.5)
7	(0.03, 0.11)	(−0.7, 2.2)
8	(0.03, 0.13)	(−0.7, 3.0)
9	(0.03, 0.15)	(−0.7, 3.7)
