# Supplementary material for: Sparse Sensing and Machine Learning for Rapid Calibration of Defect Detection with rf Atomic Magnetometers
Source: Sensors (Basel). 2025 Nov 13;25(22):6930. doi: 10.3390/s25226930 (PMC12656515; doi:10.3390/s25226930)
Supplement: Supplementary file 1 [file sensors-25-06930-s001.zip › sensors-3920812-supplementary.pdf]

Sparse sensing and machine learning for rapid calibration of defect detection with rf atomic magnetometers: supplementary material

M. J. Munko[1], L. M. Rushton[2], L. M. Ellis[2], J. D. Zipfel[2], P. Bevington[2], W. Chalupczak[2], S. Lopez Dubon[1]

[1] School of Engineering, The University of Edinburgh, Mayfield Road, Edinburgh, EH9 3JL, United Kingdom; [2] National Physical Laboratory, Hampton Road, Teddington, TW11 0LW, United Kingdom

1 Phase Response

The phase describes a change in direction of B_{rf} with respect to B_0, e.g. the projection axis. Taken for the calibration data set as a whole, Fig. A.1 (b), there is a vortex in the phase data; i.e. for a fixed spatial position, and therefor secondary field direction, the phase data describes the change angle of the projection of the measured rf field on to the sensing plane, as the sensing plane rotates. For spatial scans, the phase change in each sub-phase-image describes the change in the direction in the secondary field around recess/edge of the plate. At the calibration point the change in secondary field describes a vortex, Fig. A.1 (a). For large angles, when there is a greater contribution of primary field in the measurement, the phase change is reduced. Approaching angles of 90°, the insensitive axis would be in the transverse plane and no secondary field would be measured in this direction.

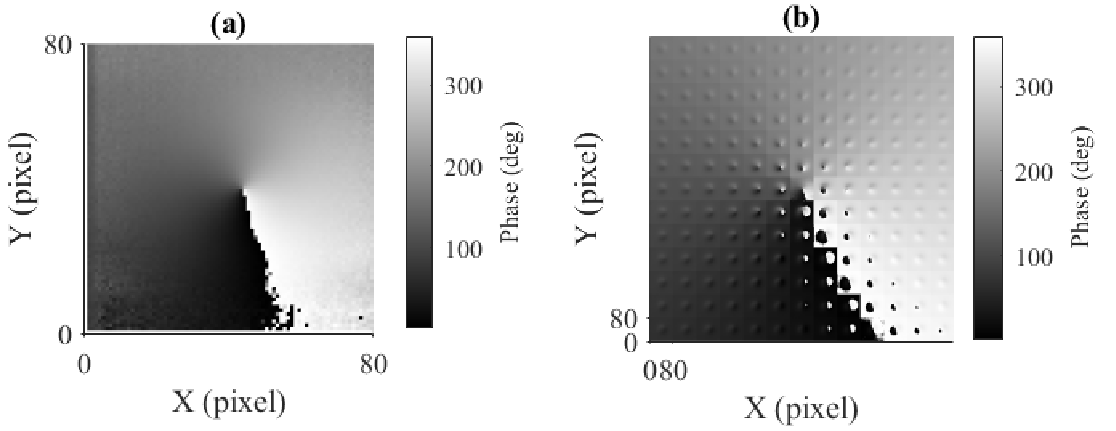

Figure A.1: (a) Image of the phase response for the calibrated measurement and for (b) the full calibration training data set, shown in Fig. 2 (b) in the main text.

2 Experimental Setup

Photographs of the experimental setup are shown in Fig A.2, where (a) shows a close up of the object, rf coil, and position of the vapour cell and fluxgate, while (b) shows the nested Helmholtz coil set and the translation stage. Figure A.2(b) is taken with a wide-angle lens to show greater detail of the setup.

3 Compressed and Tailored Sensing

This section aims to introduce the mathematical preliminaries for sensing location optimisation and high-resolution image reconstruction. To aid the understanding of the process, the key mathematical symbols are

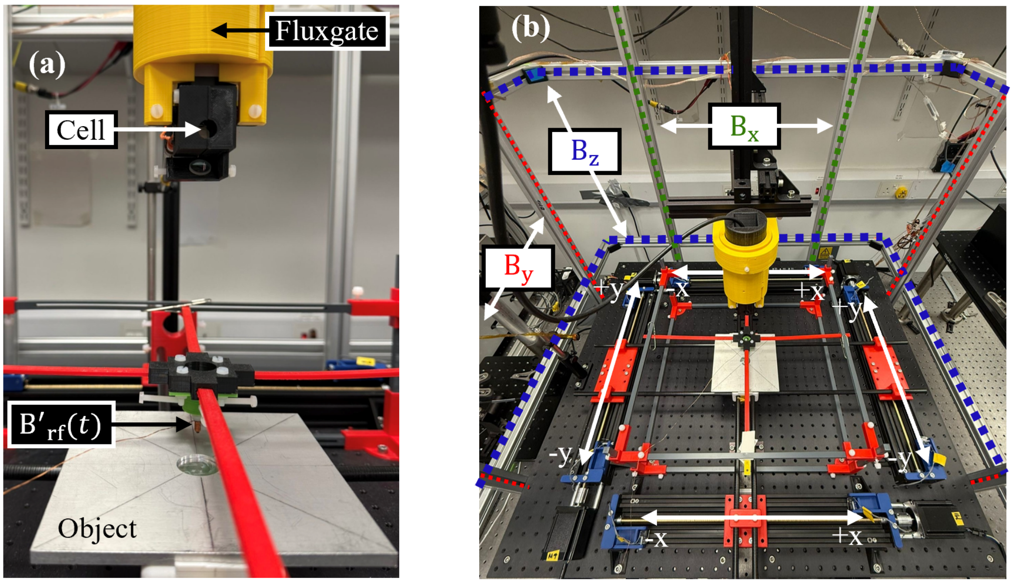

Figure A.2: Images of the experimental setup with scanning rig showing: (a) the object (aluminium plate with a 2.4-mm-deep and 24-mm-diameter recess), the rf coil used to generate $\mathbf{B}'_{\mathrm{rf}}(t)$, and the position of the vapour cell and fluxgate (used to monitor $\mathbf{B}_0$), both enclosed in a 3D-printed black and yellow holder; (b) the three orthogonal nested square coil array used to generate $\mathbf{B}_0$ (field coil pairs and relative field direction marked with white arrows) and the pairs of stepper motors used for translating the object in the along the x- and y-axes (translation direction marked with white arrows).

summarised in Table 1.

3.1 Optimising sensing locations

3.1.1 Compressed sensing

The fundamental idea of compressed sensing is the ability to leverage the sparsity of a real-world signal to reconstruct its full-resolution representation using a limited number of measurements [1]. According to Mahonar et al. [1], any single high-dimensional system measurement, $\mathbf{x}$, can be represented as

$$\mathbf{x} = \boldsymbol{\psi}\mathbf{s}, \tag{A.1}$$

where $\boldsymbol{\psi}$ is the universal transform basis, such as a matrix of Fourier or wavelet coefficients, and $\mathbf{s}$ is a sparse vector. In this way, a sparse signal $\mathbf{s}$ can be used to reconstruct high-dimensional information $\mathbf{x}$, according to the principle of compressed sensing. This can be further represented as

$$\mathbf{y} = \mathbf{C}\boldsymbol{\psi}\mathbf{s}, \tag{A.2}$$

where $\mathbf{y}$ is a vector of system measurements and $\mathbf{C}$ indicates the location of sensors. Compressed sensing can be performed efficiently when the location of sensors, $\mathbf{C}$, is chosen at random [1].

3.1.2 Tailored sensing

While compressed sensing aims to reconstruct a high-dimensional signal using a limited number, r, of measurements using a universal transform basis, $\boldsymbol{\psi}$, tailored sensing takes this idea a step further, incorporating the use of a system-specific basis. The system-specific basis, $\boldsymbol{\psi}_r$, can be constructed using historic measurements of the system, allowing the sensing locations to be tailored to improve the quality of reconstruction. According to [3], Eq. A.2 can be rewritten as

$$\mathbf{y} = \mathbf{C}\boldsymbol{\psi}_r\mathbf{a}, \tag{A.3}$$

where $\mathbf{a}$ is the vector used for reconstruction which, unlike $\mathbf{s}$, is no longer sparse, but has the same dimensions as $\mathbf{y}$.

One of the ways to create a dataset-specific basis is by performing singular value decomposition (SVD) on the collected historical data [1]. Such a dataset can be represented as a matrix $\mathbf{X}$, where each column is a single high-dimensional measurement $\mathbf{x}$, flattened into a one-dimensional vector. Therefore, if each measurement $\mathbf{x}$

Table 1: Summary of Symbols Used

**Type**	**Explanation**
Scalars	
r	Number of optimized sensing locations
m	Number of pixels in each measurement
n	Number of measurements in the dataset
1D Vectors	
$\mathbf{x} \in \mathbb{R}^{m\times 1}$	Full-resolution system measurement
$\mathbf{s} \in \mathbb{R}^{m\times 1}$	Sparse reconstruction vector
$\mathbf{y} \in \mathbb{R}^{r\times 1}$	Tailored system measurements
$\mathbf{a} \in \mathbb{R}^{r\times 1}$	Tailored basis coefficients
$\mathbf{x}_{\text{rec}} \in \mathbb{R}^{m\times 1}$	Reconstructed full-resolution measurement
Matrices	
$\Psi \in \mathbb{R}^{m\times m}$	Universal transform basis
$\Psi_r \in \mathbb{R}^{m\times r}$	Dataset-specific transform basis approximation
$\mathbf{C} \in \mathbb{R}^{r\times m}$	Sensing location matrix
$\mathbf{X} \in \mathbb{R}^{m\times n}$	Dataset of flattened system measurements
$\mathbf{U} \in \mathbb{R}^{m\times m}$	Left singular vectors (SVD product)
$\Sigma \in \mathbb{R}^{m\times n}$	Singular values (SVD product)
$\mathbf{V} \in \mathbb{R}^{n\times n}$	Right singular vectors (SVD product)
$\mathbf{Q} \in \mathbb{R}^{m\times m}$	Orthonormal matrix (QR factorisation product)
$\mathbf{R} \in \mathbb{R}^{m\times n}$	Upper triangular matrix (QR factorisation product)

has m dimensions, and n measurements have been recorded, $\mathbf{X}$ would have a size of $m \times n$. SVD is an operation decomposing $\mathbf{X}$ into a product of three matrices: $\mathbf{U}$ representing left singular vectors, $\mathbf{\Sigma}$ containing singular values on its diagonal, and $\mathbf{V}^T$ containing right singular vectors [2]:

$$\mathbf{X} = \mathbf{U\Sigma V}^{\mathrm{T}}. \tag{A.4}$$

The component of particular relevance to creating a dataset-specific basis is $\mathbf{U}$. Its columns are orthonormal vectors that can be interpreted as high-level features of the original dataset, and each of the entries of $\mathbf{X}$ can be reconstructed through a linear combination of the columns of $\mathbf{U}$. Although the maximum size of $\mathbf{U}$ is equal to the original size of $\mathbf{X}$, an approximation of the original dataset can be obtained by considering r leftmost columns of $\mathbf{U}$, where $r < n$ and $r \in \mathbb{Z}$. Therefore, the approximated SVD result can be rewritten as

$$\mathbf{X} \cong \mathbf{U}_r \mathbf{\Sigma}_{\boldsymbol{r}} \mathbf{V}_r^{\mathrm{T}}. \tag{A.5}$$

Considering the approximated dataset-specific basis, it has been observed that optimal sensing locations can be found when pivoted QR decomposition is performed on $\mathbf{U}_r$ [1]. The operation decomposes the input matrix into a product of $\mathbf{Q}$ and $\mathbf{R}$, where $\mathbf{Q}$ is orthonormal, and $\mathbf{R}$ is an upper triangular matrix. When column pivoting is used, i.e., columns are reordered in the computation process to improve QR factorisation, the pivot locations coincide with the optimal sensing locations for the system. Therefore, pivoted QR decomposition can be used to determine the sensing locations $\mathbf{C}$ shown in Eq. A.3. Once $\mathbf{C}$ is computed, the measurements of the system, $\mathbf{y}$, can be made at the specific locations. Once $\mathbf{y}$, $\mathbf{C}$, and $\boldsymbol{\psi}_r$ are known, $\mathbf{a}$ can be determined, as it can be deduced from Eq. A.3. Subsequently, $\mathbf{a}$ can be used to find the full-resolution reconstruction $\mathbf{x}_{\text{rec}}$, using the following relationship:

$$\mathbf{x}_{\text{rec}} = \boldsymbol{\psi}_r \mathbf{a}. \tag{A.6}$$

The steps required to optimise sensing locations based on the collected training dataset, as well as the full-resolution image reconstruction method, are presented in the pseudocode format in Section 3.2.

3.2 Pseudocode representation for the processes

Algorithms 1 and 2 are pseudocode representations of the computational processes used in this work. Algorithm 1 shows the steps required to compute optimal sensing locations for a list of different sensor numbers considered, r_{list}, using a training dataset, $\mathbf{X}$.

Algorithm 1 Sensing location optimisation

Require: Training dataset $\mathbf{X} \in \mathbb{R}^{m \times n}$, list of sensing location numbers to examine r_{list}
Compute SVD on $\mathbf{X}$ to obtain $\mathbf{U}$, $\mathbf{\Sigma}$, $\mathbf{V}^T$
for r in r_{list} **do**
  Set $\boldsymbol{\psi}_r$ to the first r columns of $\mathbf{U}$
  Compute pivoted QR factorisation on $\boldsymbol{\psi}_r$
  Store pivot locations in a sparse matrix C
end for
return Optimised sensing locations C for each r considered

Algorithm 2 shows the way to reconstruct a full-resolution sample when the optimised sensing locations $\mathbf{C}$ and the dataset-specific basis $\boldsymbol{\psi}_r$ are known.

Algorithm 2 Reconstruction of a single full-resolution image

Require: Dataset-specific basis $\boldsymbol{\psi}_r$, optimised sensing locations $\mathbf{C}$
Obtain system readings $\mathbf{y}$ at locations $\mathbf{C}$
Multiply $\mathbf{C}$ by $\boldsymbol{\psi}_r$ and store it as $\boldsymbol{\theta}$
Compute the inverse of $\boldsymbol{\theta}$
Multiply $\boldsymbol{\theta}^{-1}$ by $\mathbf{y}$ to compute $\mathbf{a}$
Multiply $\boldsymbol{\psi}_r$ by $\mathbf{a}$ to compute the reconstruction $\mathbf{x}_{\text{rec}}$
Reshape $\mathbf{x}_{\text{rec}}$ from a flat vector to a two-directional matrix $\mathbf{x}_{\text{rec2D}}$
return High-dimensional image reconstruction $\mathbf{x}_{\text{rec2D}}$

4 Image downsampling

The resolution of each scan in the original measurement, presented in the original raster scan mosaic, is 80×80 pixels. This resolution was chosen as it was considered to be the maximum resolution in which a 13×13 mosaic could have been recorded in a feasible time. However, it can be observed that satisfactory results can also be achieved with a 40×40 pixel scan, relative to the original 80×80 measurement. The downsampling presented in this work is performed by dividing each 80×80 pixel image into 2×2 pixel blocks and finding their average. The reduction in the number of measured pixels by a factor of four is the first step in reducing the data redundancy of the collected images.

The Peak Signal-to-Noise Ratio (PSNR) has been computed for both dataset resolutions. It has been found that the distribution of PSNR across samples is in good agreement between the two datasets, with a Pearson's correlation coefficient equal to 99.999%. The mean PSNR for the original dataset is 18.31, and for the downsampled dataset, 18.37, indicating a 0.28% improvement. However, the visual inspection by a skilled observer is indispensable to determine if the characteristic features in the image have been preserved.

Further reduction in the resolution of the image is not considered. The main motivation for the downsampling process has been to reduce the time needed to record a new dataset, and it is believed that collecting a dataset of 40 x 40 images is feasible (resulting in a four-times speed-up relative to the original dataset). While an additional decrease in the resolution of the image might impact the quality of characteristic features, it is not thought to significantly reduce the number of sensing locations required for reconstruction. The importance of sensing pixels is high as they bring in new information about the system in critical locations, while the remaining pixels are often highly correlated.

References

[1] Manohar, K.; Brunton, B.W.; Kutz, J.N.; Brunton, S.L. Data-Driven Sparse Sensor Placement for Reconstruction: Demonstrating the Benefits of Exploiting Known Patterns. *IEEE Control Syst. Mag.* **2018**, *38*(3), 63–86.

[2] Wall, M.E.; Rechtsteiner, A.; Rocha, L.M. Singular Value Decomposition and Principal Component Analysis. In: Berrar, D.P.; Dubitzky, W.; Granzow, M. (Eds.) *A Practical Approach to Microarray Data Analysis*; Springer: Boston, MA, USA, 2003.

[3] Manohar, K.; Hogan, T.; Buttrick, J.; Banerjee, A.G.; Kutz, J.N.; Brunton, S.L. Predicting Shim Gaps in Aircraft Assembly with Machine Learning and Sparse Sensing. *J. Manuf. Syst.* **2018**, *48*, Part C, 87–95.

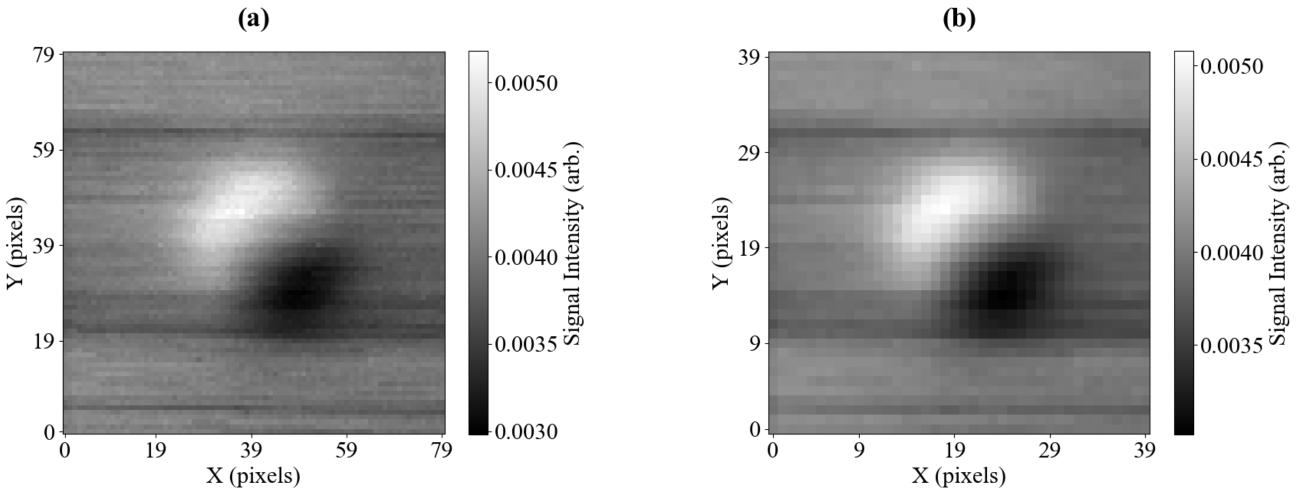

Figure A.3: (a) A chosen 80×80 pixel sample from the original dataset. (b) The 40×40 representation of the sample shown in (a) following a downsampling process.
